# Supplementary material for: Risk factors for the development of hepatocellular carcinoma (HCC) in chronic hepatitis B virus (HBV) infection: a systematic review and meta‐analysis
Source: J Viral Hepat. 2020 Dec 28;28(3):493–507. doi: 10.1111/jvh.13452 (PMC8581992; doi:10.1111/jvh.13452)
Supplement: Supplementary file 1 — Supplementary Material [file JVH-28-493-s001.docx]

**Appendices (**available online at Figshare (10.6084/m9.figshare.12668351)**)**

PRISMA Checklist

| **Section/topic** | **#** | **Checklist item** | **Reported in section** |
| --- | --- | --- | --- |
| **TITLE** | | |  |
| Title | 1 | Identify the report as a systematic review, meta-analysis, or both. | Abstract |
| **ABSTRACT** | | |  |
| Structured summary | 2 | Provide a structured summary including, as applicable: background; objectives; data sources; study eligibility criteria, participants, and interventions; study appraisal and synthesis methods; results; limitations; conclusions and implications of key findings; systematic review registration number. | Abstract |
| **INTRODUCTION** | | |  |
| Rationale | 3 | Describe the rationale for the review in the context of what is already known. | 1. |
| Objectives | 4 | Provide an explicit statement of questions being addressed with reference to participants, interventions, comparisons, outcomes, and study design (PICOS). | 1. |
| **METHODS** | | |  |
| Protocol and registration | 5 | Indicate if a review protocol exists, if and where it can be accessed (e.g., Web address), and, if available, provide registration information including registration number. | N/A |
| Eligibility criteria | 6 | Specify study characteristics (e.g., PICOS, length of follow-up) and report characteristics (e.g., years considered, language, publication status) used as criteria for eligibility, giving rationale. | 2.1 |
| Information sources | 7 | Describe all information sources (e.g., databases with dates of coverage, contact with study authors to identify additional studies) in the search and date last searched. | 2.1 |
| Search | 8 | Present full electronic search strategy for at least one database, including any limits used, such that it could be repeated. | 2.1, supplement |
| Study selection | 9 | State the process for selecting studies (i.e., screening, eligibility, included in systematic review, and, if applicable, included in the meta-analysis). | 2.1 |
| Data collection process | 10 | Describe method of data extraction from reports (e.g., piloted forms, independently, in duplicate) and any processes for obtaining and confirming data from investigators. | 2.2 |
| Data items | 11 | List and define all variables for which data were sought (e.g., PICOS, funding sources) and any assumptions and simplifications made. | 2.2 |
| Risk of bias in individual studies | 12 | Describe methods used for assessing risk of bias of individual studies (including specification of whether this was done at the study or outcome level), and how this information is to be used in any data synthesis. | 2.3 |
| Summary measures | 13 | State the principal summary measures (e.g., risk ratio, difference in means). | 2.2 |
| Synthesis of results | 14 | Describe the methods of handling data and combining results of studies, if done, including measures of consistency (e.g., I^2^) for each meta-analysis. | 2.2 |

| **Section/topic** | **#** | **Checklist item** | **Reported on page #** |
| --- | --- | --- | --- |
| Risk of bias across studies | 15 | Specify any assessment of risk of bias that may affect the cumulative evidence (e.g., publication bias, selective reporting within studies). | N/A |
| Additional analyses | 16 | Describe methods of additional analyses (e.g., sensitivity or subgroup analyses, meta-regression), if done, indicating which were pre-specified. | 2.2 |
| **RESULTS** | | |  |
| Study selection | 17 | Give numbers of studies screened, assessed for eligibility, and included in the review, with reasons for exclusions at each stage, ideally with a flow diagram. | 3.1 |
| Study characteristics | 18 | For each study, present characteristics for which data were extracted (e.g., study size, PICOS, follow-up period) and provide the citations. | Supplement |
| Risk of bias within studies | 19 | Present data on risk of bias of each study and, if available, any outcome level assessment (see item 12). | 3.2, Supplement |
| Results of individual studies | 20 | For all outcomes considered (benefits or harms), present, for each study: (a) simple summary data for each intervention group (b) effect estimates and confidence intervals, ideally with a forest plot. | 3.3, 3.4 |
| Synthesis of results | 21 | Present results of each meta-analysis done, including confidence intervals and measures of consistency. | 3.3.1 |
| Risk of bias across studies | 22 | Present results of any assessment of risk of bias across studies (see Item 15). | N/A |
| Additional analysis | 23 | Give results of additional analyses, if done (e.g., sensitivity or subgroup analyses, meta-regression [see Item 16]). | 3.3.2 |
| **DISCUSSION** | | |  |
| Summary of evidence | 24 | Summarize the main findings including the strength of evidence for each main outcome; consider their relevance to key groups (e.g., healthcare providers, users, and policy makers). | 4.1 |
| Limitations | 25 | Discuss limitations at study and outcome level (e.g., risk of bias), and at review-level (e.g., incomplete retrieval of identified research, reporting bias). | 4.2, 4.3, 4.6 |
| Conclusions | 26 | Provide a general interpretation of the results in the context of other evidence, and implications for future research. |  |
| **FUNDING** | | |  |
| Funding | 27 | Describe sources of funding for the systematic review and other support (e.g., supply of data); role of funders for the systematic review. |  |

*From:*  Moher D, Liberati A, Tetzlaff J, Altman DG, The PRISMA Group (2009). Preferred Reporting Items for Systematic Reviews and Meta-Analyses: The PRISMA Statement. PLoS Med 6(7): e1000097. doi:10.1371/journal.pmed1000097. For more information, visit: **www.prisma-statement.org**.

**Table S1**. Search terms used in systematic search of Embase, MEDLINE and Web of Science databases.

| **Criteria** | **Database** | **Search terms** |
| --- | --- | --- |
| Population | MEDLINE and Embase (searched via Ovid) | ("hepatitis b" or "hbv" or "hepatitis").ab. |
|  | Web of Science | AB=("Hepatitis B" or "hbv" or ("hepatitis" and "b")) |
| Exposure | MEDLINE and Embase (searched via Ovid) | ("diabetes mellitus" or "diabetes" or "hypertensi*" or "hyperlipidaemia" or "hyperlipidemia" or "dyslipidaemia" or "dyslipidemia" or "obes*" or "overweight" or "osteoporosis" or "osteopenia" or "osteopaenia" or "renal disease" or "kidney disease" or "vitamin d" or "comorb*" or "comorbidit*").ab. |
|  | Web of Science | AB=(“diabetes mellitus” or “diabetes” or “hypertensi*” or "hypertensi*" or "hyperlipidaemia" or "hyperlipidemia" or "dyslipidaemia" or "dyslipidemia" or "obes*" or "overweight" or "osteoporosis" or "osteopenia" or "osteopaenia" or "renal disease" or "kidney disease" or "vitamin d" or "comorb*" or "comorbidit*") |
| Outcome | MEDLINE and Embase (searched via Ovid) | ("hepatocellular carcinoma" or "hcc" or "hepatoma" or ("hepatocellular" and "carcinoma")).ab. |
|  | Web of Science | AB=(“hepatocellular carcinoma” or “hcc” or “hepatoma” or (“hepatocellular” and “carcinoma”)) |
| Final search | MEDLINE and Embase (searched via Ovid) | (("hepatitis b" or "hbv" or "hepatitis").ab.) AND (("diabetes mellitus" or "diabetes" or "hypertensi*" or "hyperlipidaemia" or "hyperlipidemia" or "dyslipidaemia" or "dyslipidemia" or "obes*" or "overweight" or "osteoporosis" or "osteopenia" or "osteopaenia" or "renal disease" or "kidney disease" or "vitamin d" or "comorb*" or "comorbidit*").ab.) AND (("hepatocellular carcinoma" or "hcc" or "hepatoma" or ("hepatocellular" and "carcinoma")).ab.) |
|  | Web of Science | (AB=("Hepatitis B" or "hbv" or ("hepatitis" and "b"))) AND (AB=(“diabetes mellitus” or “diabetes” or “hypertensi*” or "hypertensi*" or "hyperlipidaemia" or "hyperlipidemia" or "dyslipidaemia" or "dyslipidemia" or "obes*" or "overweight" or "osteoporosis" or "osteopenia" or "osteopaenia" or "renal disease" or "kidney disease" or "vitamin d" or "comorb*" or "comorbidit*")) AND (AB=(“hepatocellular carcinoma” or “hcc” or “hepatoma” or (“hepatocellular” and “carcinoma”))) |
| MH, medical subject heading term.  Search results were restricted to human studies published in English from 2000 to 2020. | | |

**Table S2.** Summary characteristics of included observational studies.

| **Country, Author (Year)** | **Study design** | **Population** | **Median follow-up period, years** | **Risk factors**  **investigated** | **Participants, n** | **HCC Cases, n** | **Age at baseline, years** | **Sex (% male)** | **Covariate adjustment** |
| --- | --- | --- | --- | --- | --- | --- | --- | --- | --- |
| UK, Ferreira (2020) ^1^ | Retrospective cohort study | CHB | 8.7§ (DM)  6.2§ (non-DM) | T2DM | 3927 | 16-19 | 39.2 (mean, non-DM)  54.9 (mean, DM) | 50 | Age,  BMI,  Cigarette smoking,  Calendar year,  Dyslipidemia,  Ethnicity,  HBV vaccination,  HCV coinfection,  Other liver disease (excluded),  Sex |
| Korea, Goh (2020) ^2^ | Retrospective cohort study | CHB patients undergoing liver biopsy from one hospital | 7.2 | Cholesterol >200mg/dL,  DM (type unspecified),  HT,  Statin use | 7713 | 702 | 50 (statin users)  47 (statin nonusers) | 66 | Age,  ALT,  Antiplatelet therapy,  Antiviral therapy,  Any other malignancy (excluded),  Cirrhosis,  DM,  HBV DNA,  HCV coinfection (excluded),  HIV coinfection (excluded),  Sex |
| Korea, Kim (2020) ^3^ | Prospective cohort study | CHB patients treatment-naïve at baseline | 4.9 | DM (type unspecified) | 3277 | 292 | 48.7 (mean) | 63 | Age,  Cirrhosis,  Compromised liver  function (excluded),  HCV coinfection,  Other significant medical illness (excluded),  Sex, |
| Singapore, Lim (2020) ^4^ | Retrospective cohort study | CHB patients undergoing liver biopsy from one hospital | 9.3 | T2DM | 289 | 27 | 43.2 (mean, Nonhepatic steatosis)  46.4 (mean, Hepatic steatosis) | 72 | Age,  Autoimmune disease,  HCV coinfection (excluded),  HIV coinfection (excluded),  Metabolic factors,  Primary biliary cirrhosis (excluded),  Primary sclerosing cholangitis (excluded),  Steatosis-inducing medication use (excluded),  Substantial alcohol  intake (excluded) |
| United States and Asia-Pacific, Yang (2020) ^5^ | Prospective cohort study | Treated CHB patients | 5.6§ | DM (type unspecified) | 5365 | 378 | 48.4 (mean) | 69 | AFP,  Age,  Albumin,  Alcohol use,  ALT,  Ascites or encephalopathy,  Bilirubin,  Cirrhosis,  HBeAg,  HCV coinfection  (excluded),  HDV coinfection  (excluded),  HIV coinfection  (excluded),  Immunosuppressant use  (excluded),  Platelet count  Sex |
| Hong Kong, Yip (2020) ^6^ | Retrospective cohort study | CHB patients with DM | 7.1 | Aspirin/clopidogrel use,  Insulin use,  Metformin use,  Statin use,  Sulphonylurea use,  Thiazolidinedione use | 28,999 | 2,655 | 57.7 (mean) | 61 | Age,  Albumin,  ALT,  Antiviral treatment,  Autoimmune disease  (excluded),  Bilirubin,  Cirrhosis,  Diabetes,  HbA1c,  HCV coinfection  (excluded),  HDV coinfection  (excluded),  HIV coinfection  (excluded),  Hypertension,  Other metabolic liver disease (excluded),  Platelets,  Renal disease  (excluded),  Sex |
| France, Brichler (2019) ^7^ | Prospective cohort study | Cirrhotic CHB patients | 5.4 | HT,  Obesity (BMI ≥ 30 kg/m^2^) | 317 | 27 | 53 (median) | 82 | Age,  HCV coinfection  (excluded),  Platelets |
| Korea, Cho (2019) ^8^ | Retrospective cohort study | Treated CHB patients | 3.6 | CVD,  DM (type unspecified),  Dyslipidaemia,  HT,  NAFLD, | 826 | 86 | 52 (mean, NAFLD)  54 (mean, non-NAFLD) | 61 | Age,  Autoimmune liver  disease (excluded),  Cirrhosis,  Compromised liver  function (excluded),  HBeAg,  HBV DNA,  Other liver disease  (excluded),  Other viral infections  (excluded),  Steatosis-inducing medication use (excluded),  Substantial alcohol  intake (excluded) |
| Taiwan, Shyu (2019) ^9^ | Nested case-control | Treated and untreated CHB patients | 11.4 | DM (type unspecified) | 5,932 | 731 | 40-90 (range) | 56.7 | Age (matched),  Alcohol-related liver disease (matched),  Antiviral treatment (excluded),  Baseline liver cirrhosis (matched),  Deyo-Charlson comorbidity index^10^†,  HCV coinfection (excluded),  Obese/overweight,  Sex (matched) |
| China, Tan (2019) ^11^ | Prospective cohort study | CHB patients treatment-naïve at baseline | 6.3§ | HT  Hypercholesteraemia,  Hypertriglyceridaemia,  Obesity (> 30kg/m^2^),  T2DM | 4454 | 89 | 45.4 (mean) | 68 | Age,  Alcohol consumption,  Antiviral therapy  (excluded),  Any other malignancy,  AST (≥40 U/L),  Cigarette smoking,  Cirrhosis,  HCV coinfection  (excluded),  High HBV viral load  (excluded),  HIV coinfection  (excluded),  Sex, |
| China, Wang (2019) ^12^ | Prospective cohort study | CHB patients treatment-naïve at baseline | 4.1 | DM (type unspecified) | 1325 | 105 | 50 (median) | 73 | Age,  Fibrosis,  HCV coinfection  (excluded),  HIV coinfection (excluded),  Immunosuppressant therapy (excluded),  Low HBV viral load (excluded),  Other liver disease  (excluded) |
| Taiwan, Hong Kong, Hsu (2018) ^13^ | Retrospective cohort study | CHB patients receiving ETV or TDF | 2.2 (Taiwan)  2.8 (Hong Kong) | DM (I and II) | 23,851 (Taiwan)  19,321 (Hong Kong) | 596 (Taiwan)  383 (Hong Kong) | 47.5  (median, Taiwan)  52.1 (median, Hong Kong) | 74 (Taiwan)  66.05 (Hong Kong) | Age,  Antiviral treatment (excluded),  Cirrhosis,  DM,  HCV coinfection (excluded),  Sex |
| Taiwan, Hsu (2018) ^14^ | Retrospective cohort study | CHB patients receiving ETV or TDF for ≥ 3 months | 2.1 | HT,  DM (I and II) | 27,820 | 802 | 48.1 (median) | 74 | Age,  Antiviral treatment (excluded),  Cirrhosis,  DM,  HCV,  Hyperlipidaemia,  Hypertension,  Interferon use,  Metformin use,  Sex,  Statin use |
| US, Kennedy (2018) ^15^ | Case-control (3:1 ratio) | Cases and controls selected from two independent medical centres | - | DM (I and II) | 1,101 | 278 | 64 | 78 | Age (matched),  Alcohol misuse,  Cirrhosis,  DM,  Ethnicity,  HCV,  HIV,  Sex (matched) |
| Korea, Kim (2018) ^16^ | Retrospective cohort study | Treated and untreated male CHB patients | 8.0 years‡ | T2DM | 214,167 | 11,241 | N/A | 100 | Age,  Alcohol consumption,  Alcohol liver disease,  ALT,  Antiviral therapy,  Any other malignancies,  Aspirin use,  BMI,  Cerebrovascular disease,  Chronic pulmonary disease,  Cigarette smoking,  Cirrhosis,  Congestive heart failure,  dementia,  HCV,  Hemiplegia or paraplegia,  HIV/AIDS,  HT,  Insurance premium,  Metastatic solid tumour,  Metformin use,  Myocardial infarction,  NAFLD,  NSAID use,  Peptic ulcer disease,  Peripheral vascular disease,  Physical activity,  Renal disease,  Residential area,  Rheumatic disease,  Sex (restricted to males),  Total cholesterol level |
| China, Li (2018) ^17^ | Case-control | Cases and controls selected from one hospital | - | DM (type unspecified) | 322 | 112 | 52 (median, HCC cases),  51(median, non-HCC controls) | 18.8 (HCC cases)  26.2 (non-HCC controls) | Age (matched),  Ant other malignancies (excluded),  Autoimmune hepatitis (excluded),  Cigarette smoking,  Cirrhosis,  Family history of HCC,  HBV DNA,  HCV (excluded),  HIV (excluded),  NAFLD (excluded),  Sex (matched) |
| Hong Kong, Yip (2018) ^18^ | Retrospective cohort study | CHB patients who have achieved HBsAg seroclearance | 3.4 | DM (type unspecified),  Statin use | 4568 | 54 | 56.7 (mean) | 63 | Age,  Antiviral treatment use prior to seroclearance,  Cirrhosis,  HCV coinfection  (excluded),  HDV coinfection  (excluded),  Liver transplant  (excluded),  Sex,  Statin use |
| Hong Kong, Chan  (2017) ^19^ | Retrospective cohort study | CHB patients undergoing liver biopsy from one hospital | 6.7 | DM (type unspecified),  HT | 270 | 11 | 43.6 (mean) | 75.2 | No adjustments |
| US, Chayanupatkul (2017) ^20^ | Retrospective  Cohort study | CHB patients attending Veterans Affairs hospitals | 4.7 years§ | DM (type unspecified),  HT¶,  Hyperlipidaemia††  IHD††,  Obesity (BMI > 30 kg/m^2^) ¶ | 8539 | 317 | N/A | N/A | Age.  ALT,  Cirrhosis (excluded),  Family history of HCC,  HCV coinfection,  Race |
| China, Han (2017) ^21^ | Case-control | Cirrhotic patients with one positive HBsAg | - | T2DM | 182 | 73 | 56.18 (mean) | 79.1% | Age (matched),  Any other malignancies (excluded),  Child-Pugh score (matched),  Sex (matched) |
| Korea, Kim (2017) ^22^ | Retrospective  cohort study | CHB patients not on treatment | 5.0 | Insulin resistance (estimated using serum glucose),  HT | 1696 | 24 | 50 (median) | 56.8 | Age,  Cirrhosis,  DM,  HBV DNA levels,  HCV coinfection (excluded),  HIV coinfection (excluded),  Obesity,  Sex |
| France, Mallet (2017) ^23^ | Retrospective  cohort study | CHB patients | 5.0‡ | CVD,  DM (type unspecified),  Renal disease (off dialysis, no transplant),  Renal disease (on dialysis, no transplant),  Respiratory disease | 48,189 | 3145 | 44 (median) | 59 | Age,  Alcohol consumption,  Any other malignancy,  HCV coinfection,  HDV coinfection,  HIV coinfection,  Immunosuppressant use (excluded),  Obesity,  Other causes of cirrhosis,  Residency area,  Sex |
| Korea, Shim (2017) ^24^ | Retrospective  cohort study | CHB patients treatment-naïve at baseline | 3.6 | DM (type unspecified) | 356 | 45 | 52 | 60 | No adjustment |
| Taiwan, Yu (2017) ^25^ | Retrospective cohort study | Male civil servants with CHB | 19.0 | Metabolic risk factors (obesity, DM (type unspecified), hypertriglyceridemia and HT), with exposure groups split into groups of 0, 1, 2 and  ≥3 risk factors | 1690 | 158 | 48.4 (median) | 100 | Age,  Alcohol consumption,  First-degree family history of HCC,  Sex (restricted to men),  Tobacco use |
| Korea, Lee (2016) ^26^ | Retrospective cohort study | CHB patients treatment-naïve at baseline | 3.8 | Obesity (BMI ≥ 25 kg/m^2^) | 102 | 7 | 46.4 (mean) | 66 | No adjustment |
| Netherlands, Brouwer (2015) ^27^ | Retrospective cohort study | Untreated CHB patients | 10.1 | DM (type unspecified),  Obesity (BMI ≥ 27.5 kg/m^2^) | 531 | 13 | N/A for cohort | 67 | No adjustment |
| Taiwan, Fu (2015) ^28^ | Matched prospective cohort | Treated and untreated CHB patients | 5.4§ (DM)  5.5§ (non-DM) | DM (type unspecified) | 4179 | 111  42 (non-DM)  69 (DM) | 49.19 (mean, DM group)  49.05 (mean, non-DM) | 58.55 (DM),  58.37 (non-DM) | Age (matched),  Antiviral treatment,  Cirrhosis,  Deyo comorbidity index,  DM onset date (matched),  Hyperlipidaemia,  Obesity,  Sex (matched),  Statin use |
| New Zealand, Hsiang (2015) ^29^ | Retrospective cohort study | Treated and untreated CHB patients | 5.0§ | T2DM | 223 | 36 | 51 (mean) | 66.8 | Age,  Alcohol use,  Antiviral therapy initiated (yes/no),  Baseline HBV DNA,  Oesophageal varices at baseline,  HCV coinfection (excluded),  HDV infection (excluded),  HIV coinfection (excluded),  MELD score (< or >- 11),  Positive HBeAg at baseline,  Sex,  Sustained viral suppression during follow-up (yes/no),  T1DM (excluded) |
| Taiwan, Hsu (2014) ^30^ | Retrospective cohort study | Treated CHB patients with cirrhosis | 2.1 | DM (I and II),  Dyslipidaemia,  HT | 210 | 35 | 52.8 (median) | 73.3 | Age,  Antiviral therapy,  Any other malignancies (excluded),  Cirrhosis (restricted to cirrhotic patients),  HBV DNA (restricted to those with >2000 IU/mL viral load),  HCV coinfection (excluded),  HIV coinfection (excluded),  MELD score,  Previous organ transplantation,  Sex |
| South Korea, Kim (2014) ^31^ | Retrospective cohort study | Cirrhotic CHB patients treated with ETV for ≥ 12 months | 3.1 | DM (type unspecified) | 306 | 45 | 49.4 (mean) | 68.3 | Age,  Antiviral treatment (excluded untreated),  Autoimmune hepatitis (excluded),  Cirrhosis (restricted to cirrhotic patients),  Decompensated cirrhosis,  HBV DNA,  HCV coinfection (excluded),  HDV coinfection (excluded),  HIV coinfection (excluded),  No virological response at 12 months after initiation of treatment,  Serum PIIINP,  Sex |
| Taiwan, Wu (2014) ^32^ | Retrospective cohort study | Treated and untreated CHB patients | 3.5§ (treated)  5.2§ (untreated) | ACS,  Cerebrovascular disease,  COPD,  DM (type unspecified),  Hypercholesterolaemia,  Renal failure | 43190 | 5446 | 43.5 (mean, same for both treated and untreated) | 76.9 (treated),  75.5 (untreated) | Age,  Antiviral treatment,  Any other malignancies,  Cirrhosis,  DM,  HCV coinfection (excluded),  HIV coinfection (excluded),  Metformin use,  NSAIDs/aspirin use,  Sex,  Statin use |
| Taiwan, Chen (2013) ^33^ | Retrospective cohort study | CHB patients recruited from national cancer screening programme | 1-3‡ | DM (type unspecified),  Metabolic syndrome,  Obesity (BMI ≥ 27 kg/m^2^) | 5606 | 57 | >40 | N/A | No adjustment |
| China, Gao (2013) ^34^ | Cross sectional case-control (stratified by cirrhosis) | Cirrhotic CHB patients treated at a single hospital for cirrhosis and/or HCC | - | DM (type unspecified),  MAP | 370 | 122 | 54.7 (mean) | 86.9 | AFP,  Albumin,  ALT,  Any other malignancies,  Autoimmune disorders,  Bilirubin,  Cigarette smoking,  Drug- or poison-induced liver damage,  Ethnicity,  GGT,  HCV coinfection,  HDV coinfection,  Heavy alcohol consumption,  Haemoglobin,  HIV coinfection,  INR,  Platelet count,  Portal vein diameter,  Presence of ascites,  Serum sodium,  Sex |
| China, Li (2012) ^35^ | Case-control | Cases and controls selected from one hospital (untreated for CHB) | - | T2DM | 6275 | 1105 | 53.8 (mean, cases)  44.9 (mean, controls) | 84.7 (cases)  73.8 (controls) | Age,  Antiviral treatment (patients on treatment excluded),  Any other malignancies (excluded),  Child-Pugh grade of cirrhosis severity,  Cirrhosis,  Family history of HCC,  HBeAg,  HCV coinfection (excluded),  Residential area,  Sex |
| Taiwan, Chao (2011) ^36^ | Case-cohort | Male civil servants with CHB | 17.0‡ | Insulin resistance (estimated using serum glucose) | 1142 | 124 | 30-65 | 100 | Age,  Antiviral therapy (excluded),  Alcohol consumption,  BCP double mutations,  Cigarette smoking,  First-degree family history of HCC,  HBeAg,  HBV DNA,  HBV genotype,  HCV coinfection (excluded),  Number of visits |
| Australia, Walter (2011) ^37^ | Retrospective cohort | Participants with ≥ record of positive HBsAg or HBV DNA in the national notifiable diseases database | 3.0 | DM (type unspecified) | 43,892 | 242 | 34.9 (median) | N/A | Age,  Any other malignancies,  Cerebrovascular disease,  Chronic pulmonary disease,  CHF,  Dementia,  Deyo-Charlson comorbidity index^10^†,  HCV coinfection,  Peptic ulcer disease,  Peripheral vascular disease,  Renal disease,  Rheumatic disease,  Sex |
| Taiwan, Wang (2009) ^38^ | Prospective cohort study | Treated and untreated CHB patients | 8.0‡ | T2DM | 696 | 24 | 49.3 (mean) | 51.3 | Age,  Alcohol consumption,  BMI,  Cigarette smoking,  Sex |
| Taiwan, Chen (2008) ^39^ | Prospective cohort study | CHB patients recruited from national cancer screening | 12.3§ | History of DM (type unspecified),  HT | 3931 | 187 | 45.9 | 59.3 | Age,  Alcohol consumption,  BMI,  Cigarette smoking,  DM,  Educational level,  Obesity,  Sex,  Total cholesterol,  Triglycerides |
| Taiwan, Yu (2008) ^40^ | Prospective cohort study | Male civil servants with CHB (treated and untreated) | 14.7§ | DM (type unspecified) | 2903 | 134 | N/A | 100 | Age,  Alcohol consumption,  BMI,  Number of visits,  Sex (restricted to males),  Tobacco use |
| Taiwan, Lai (2006) ^41^ | Prospective cohort study | CHB patients recruited from national cancer screening | 2.8§ | T2DM | 6545 | 46 | N/A | N/A | Age,  Alcohol consumption,  Any other malignancies (excluded),  Cigarette smoking,  HCV coinfection,  Sex |
| FU, follow-up; RF, risk factors; DM, diabetes mellitus; ETV, entecavir; TDF, tenofovir disoproxil fumarate; HT, hypertension; HCV, hepatitis C virus; N/A, not available; HIV, human immunodeficiency virus; ALT, alanine aminotransferase; BMI, body mass index; NAFLD, non-alcoholic fatty liver disease; NSAID, non-steroidal anti-inflammatory drug; HCC, hepatocellular carcinoma; MELD, model for end-stage liver disease; T1DM, type 1 diabetes mellitus; HBeAg, hepatitis B E antigen; HDV, hepatitis delta virus; PIIINP, Procollagen 3 N-terminal peptide; COPD, chronic obstructive pulmonary disease; ACS, acute coronary syndrome; AFP, alpha-feto protein; GGT, gamma-glutamyl transpeptidase; INR, international normalised ratio; MAP, mean arterial pressure; BCP, basal core promoter; CHF, congestive heart failure; HBsAg, hepatitis B surface antigen.  † Comorbidity index calculated according to presence of any of the following comorbidities: myocardial infarction, congestive heart failure, peripheral vascular disease, cerebrovascular disease, dementia, chronic pulmonary disease, rheumatic disease, peptic ulcer disease, hemiplegia or paraplegia, renal disease, any malignancy, metastatic solid tumour and HIV/AIDS.  ‡ Length from index date to end of observation, as mean/median follow-up not reported in study.  § Mean length of follow-up.  ¶ Not adjusted for sex.  †† RRs for these factors not adjusted to listed covariates. | | | | | | | | | |

**Table S3**. Quality appraisal scores of case-control studies identified in meta-analysis. Quality scores awarded according to Newcastle-Ottawa Scale.

|  | **Selection** | | | | **Comparability** | **Exposure** | | |  |
| --- | --- | --- | --- | --- | --- | --- | --- | --- | --- |
| **Case-control study** | **Is case definition adequate?** | **Representativeness of cases** | **Selection of controls** | **Definition of controls** | **Comparability of cases and controls** | **Method of exposure ascertainment** | **Same method for cases and controls?** | **Non-response rate** | **Score, study quality** |
| Shyu^9^ (2019) | Yes, reference to ICD codes | Consecutive or obviously representative series of cases | Cohort controls | No history of disease | Study controls for age and sex | Secure record | Yes | Not mentioned | 7, sufficient |
| Kennedy (2018)^15^ | Yes, reference to ICD codes | Consecutive or obviously representative series of cases | Hospital controls | No history of disease | Study controls for age and sex | Secure record | Yes | Not mentioned | 6, sufficient |
| Li (2018)^17^ | Yes, reference to primary record source | Consecutive or obviously representative series of cases | Hospital controls | No mention of outcome history | Study controls for age and sex | Self report or medical record | Yes | Not mentioned | 5, sufficient |
| Han (2017)^21^ † | No | Consecutive or obviously representative series of cases | Hospital controls | No mention of outcome history | Study controls for age and sex | Medical record | Yes | Not mentioned | 4, low |
| Gao (2013)^34^ | Yes, reference to primary record source | Consecutive or obviously representative series of cases | Hospital controls | No history of disease | Study controls for sex but not age | Secure record | Yes | Same for both groups† | 7, sufficient |
| Li (2012)^35^ | Yes, reference to primary record source | Consecutive or obviously representative series of cases | Hospital controls | No mention of outcome history | Study controls for age and sex | Secure record | Yes | Same for both groups | 7, sufficient |
| Chao (2011) ^36^ | Yes, reference to primary record source | Consecutive or obviously representative series of cases | Cohort controls | No mention of outcome history | Study controls for age and sex | No description | No description | Not mentioned | 5, sufficient |
| † Participants with missing values excluded | | | | | | | | | |

**Table S4**. Quality appraisal scores of cohort studies identified in meta-analysis. Quality scores awarded according to Newcastle-Ottawa Scale.

|  | **Selection** | | | | **Comparability** | **Outcome** | | |  |
| --- | --- | --- | --- | --- | --- | --- | --- | --- | --- |
| **Cohort study** | **Representativeness of exposed cohort** | **Selection of non-exposed cohort** | **Ascertainment of exposure** | **Participants outcome-free at baseline** | **Comparability of cohorts** | **Outcome assessment** | **Sufficient length of follow-up?^‡^** | **Adequacy of follow-up** | **Score, study quality** |
| Ferreira (2020) ^1^ | Representative of average CHB in community | From same community as exposed | Secure record | Yes | Study controls for age and sex | Record linkage | No | No/minimal loss to follow up | 8, high |
| Goh (2020) ^2^ | Selected group (CHB patients undergoing liver biopsy) | From same community as exposed | Health assessment/ health record | Yes | Study controls for age and sex | Imaging assessment and pathology | No | No/minimal loss to follow up | 7, sufficient |
| Kim (2020) ^3^ | Somewhat representative of average CHB in community (restricted to patients who were treatment-naïve at baseline) | From same community as exposed | Health assessment | Yes | Study controls for age and sex | Imaging assessment and pathology | No | No statement | 7, sufficient |
| Lim (2020) ^4^ | Selected group (CHB patients undergoing liver biopsy) | From same community as exposed | Health assessment | Yes | Study controls for age | Imaging assessment | No | No/minimal loss to follow up | 6, sufficient |
| Yang (2020) ^5^ | Somewhat representative of average CHB in community (restricted to participants receiving antiviral treatment) | From same community as exposed | Health assessment/ health record | Yes | Study controls for age and sex | Cytology or pathology | No | No statement | 7, sufficient |
| Yip (2020) ^6^ | Somewhat representative of average CHB in community (restricted to CHB with DM) | From same community as exposed | Secure record | Yes | Study controls for age and sex | Record linkage | No | No/minimal loss to follow up | 8, high |
| Brichler (2019) ^7^ | Somewhat representative of average CHB in community (restricted to CHB patients with cirrhosis) | From same community as exposed | Health assessment | Yes | Study controls for age | Imaging assessment, pathology and non-invasive assessment | No | Description of patients lost to follow up | 7, sufficient |
| Cho (2019) ^8^ | Somewhat representative of average CHB in community (restricted to participants receiving antiviral treatment) | From same community as exposed | Health assessment | Yes | Study controls for age | Pathology and clinical assessment | No | Description of patients lost to follow up | 7, sufficient |
| Tan (2019) ^11^ | Somewhat representative of average CHB in community (restricted to civil servants who were treatment-naïve at baseline) | From same community as exposed | Health assessment | Yes | Study controls for age and sex | Imaging assessment and pathology | No | No/minimal loss to follow up | 8, high |
| Wang (2019) ^12^ | Somewhat representative of average CHB in community (restricted to patients who were treatment-naïve at baseline) | From same community as exposed | Health assessment/ health record | Yes | Study controls for age | Imaging assessment and pathology | No | No statement | 6, sufficient |
| Yip (2018) ^18^ | Somewhat representative of average CHB in community (restricted to patients who have achieved HBsAg seroclearance) | From same community as exposed | Secure record | Yes | Study controls for age and sex | Record linkage | No | No/minimal loss to follow up | 8, high |
| Hsu (2018) ^13^ | Somewhat representative of average CHB in community (restricted to participants receiving antiviral treatment) | From same community as exposed | Secure record | Yes | Study controls for age and sex | Independent assessment/record linkage | Yes | No/minimal loss to follow up | 9, high |
| Hsu (2018)^14^ | Somewhat representative of average CHB in community (restricted to participants receiving antiviral treatment) | From same community as exposed | Secure record | Yes | Study controls for age and sex | Record linkage | No | No/minimal loss to follow up | 8, high |
| Kim (2018)^16^ | Somewhat representative of average CHB in community (restricted to males) | From same community as exposed | Secure record | Yes | Study controls for age and sex | Record linkage | No | No/minimal loss to follow up | 8, high |
| Chan  (2017)^19^ | Selected group (CHB patients undergoing liver biopsy) | From same community as exposed | Comprehensive health assessment | Yes | None | Assessment using ≥  2 imaging techniques | No | Description of patients lost to follow up | 5, sufficient |
| Chayanupatkul (2017) ^20^ | Somewhat representative of average CHB in community (patients attending Veterans Affairs hospitals) | From same community as exposed | Secure record | Not specified | Study controls for age | Record linkage, imaging and pathology | No | No statement | 5, sufficient |
| Kim (2017) ^22^ | Representative of average CHB in community | From same community as exposed | Health assessment/ health record | Yes | Study controls for age and sex | Assessment using imaging | No | No statement | 6, sufficient |
| Mallet (2017) ^23^ | Representative of average CHB in community | From same community as exposed | Secure record | Not specified | Study controls for age and sex | Record linkage | No | No/minimal loss to follow up | 7, sufficient |
| Shim (2017) ^24^ | Somewhat representative of average CHB in community (restricted to patients who were treatment-naïve at baseline) | From same community as exposed | Secure record | Yes | None | Imaging assessment and pathology | No | No/minimal loss to follow up | 6, sufficient |
| Yu (2017)^25^ | Somewhat representative of average CHB in community (restricted to untreated males) | From same community as exposed | Health assessment/ interview | Not specified | Study controls for age and sex | Record linkage | Yes | No/minimal loss to follow up | 8, high |
| Lee (2016) ^26^ | Somewhat representative of average CHB in community (restricted to patients who were treatment-naïve at baseline) | From same community as exposed | Health assessment | Yes | None | Imaging assessment and/or biopsy | No | No/minimal loss to follow up | 6, sufficient |
| Brouwer (2015) ^27^ | Somewhat representative of average CHB in community (restricted to untreated CHB patients) | From same community as exposed | Secure record | Not specified | None | Imaging assessment and pathology | Yes | Description of patients lost to follow up | 6, sufficient |
| Fu (2015) ^28^ | Representative of average CHB in community | From same community as exposed | Secure record | Yes | Study controls for age and sex | Record linkage | No | No/minimal loss to follow up | 8, high |
| Hsiang (2015) ^29^ | Somewhat representative of average CHB in community (patients with transient elastography or radiological features of cirrhosis who sought care at a single hospital) | From same community as exposed | Health assessment/ health record | Not specified | Study controls for age and sex | Assessment using imaging | No | Description of patients lost to follow up | 7, sufficient |
| Hsu (2014) (49) | Somewhat representative of average CHB in community (restricted to participants receiving antiviral treatment) | From same community as exposed | Secure record | Yes | Study controls for age and sex | Assessment using imaging and AFP testing | No | No statement | 7, sufficient |
| Kim (2014) ^31^ | Somewhat representative of average CHB in community (restricted to cirrhotic to participants receiving antiviral treatment) | From same community as exposed | No description | Yes | Study controls for age and sex | Assessment using imaging and AFP testing | No | No/minimal loss to follow up | 7, sufficient |
| Wu (2014) ^32^ | Representative of average CHB in community | From same community as exposed | Secure record | Yes | Study controls for age and sex | Record linkage | No | No/minimal loss to follow up | 8, high |
| Chen (2013) ^33^ | Representative of average CHB in community | From same community as exposed | Health assessment | Yes | None | Record linkage | No | No/minimal loss to follow up | 6, sufficient |
| Walter (2011) ^37^ | Representative of average CHB in community | From same community as exposed | Hospital record | Yes | Study controls for age and sex | Record linkage | No | No/minimal loss to follow up | 8, high |
| Wang (2009) ^38^ | Representative of average CHB in community | From same community as exposed | Health assessment/ health record | Yes | Study controls for age and sex | Imaging and record linkage | No | Response rate of 68.4% | 7, sufficient |
| Chen (2008) ^39^ | Representative of average CHB in community | From same community as exposed | Interview | Yes | Study controls for age and sex | Record linkage | Yes | No statement | 8, high |
| Yu (2008) ^40^ | Somewhat representative of average CHB in community (restricted to males) | From same community as exposed | Health assessment/ interview | Not specified | Study controls for age and sex | Imaging | Yes | No statement | 7, sufficient |
| Lai (2006) ^41^ | Representative of average CHB in community | From same community as exposed | Health assessment | Yes | Study controls for age and sex | Imaging assessment and pathology | No | No/minimal loss to follow up | 8, high |
| CHB, chronic hepatitis B; HBsAg, hepatitis B surface antigen; AFP, alpha-fetoprotein. | | | | | | | | | |


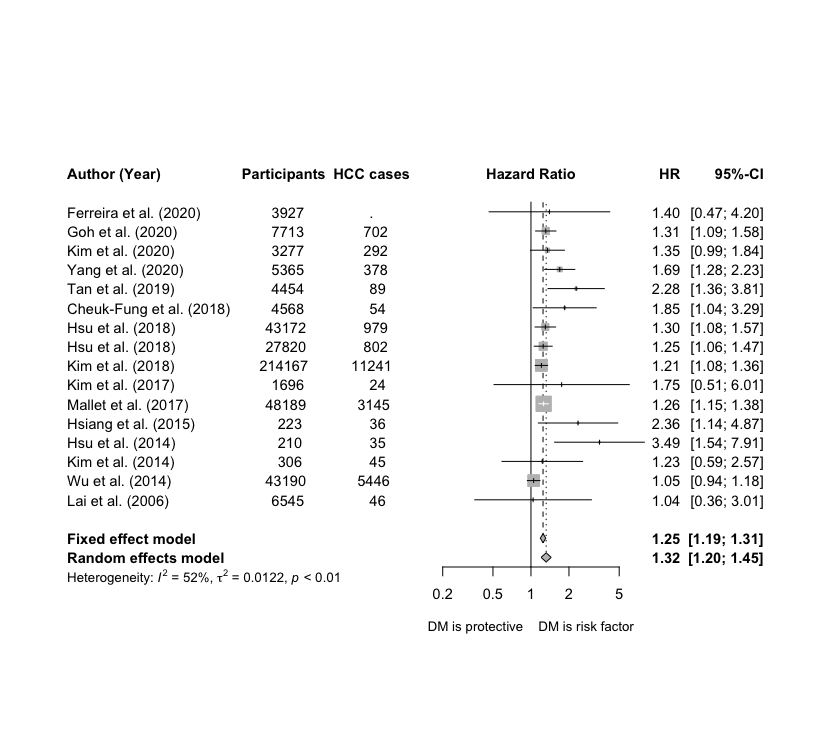


**Figure S1.** Sensitivity analysis of primary meta-analysis demonstrating the influence of restricting the analysis to studies additionally adjusting for HCV coinfection in addition to age and sex. All included studies were cohort or nested case-control studies reporting Hazards Ratios.

HR, hazard ratio; CI, confidence interval; DM, diabetes mellitus.

**
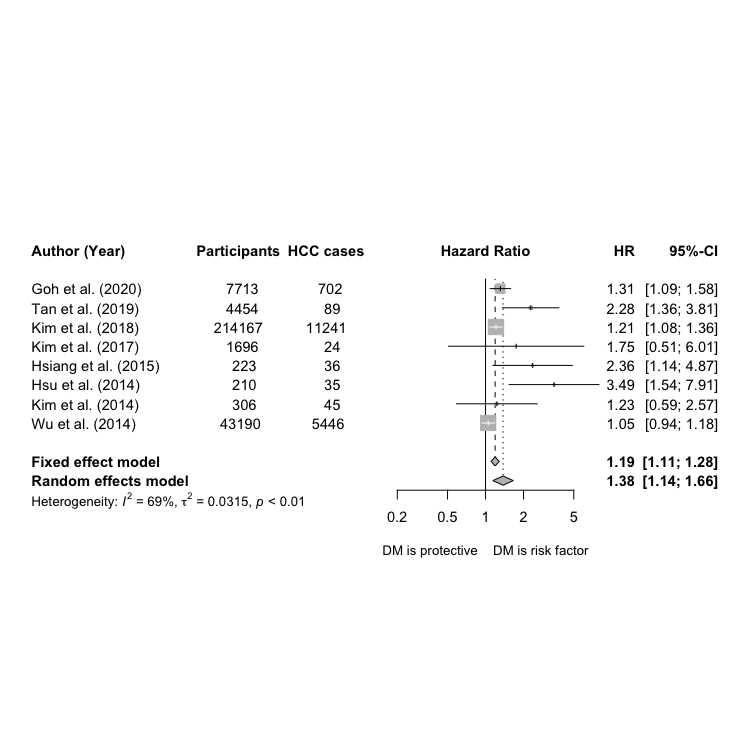
**

**Figure S2.** Sensitivity analysis of primary meta-analysis demonstrating the influence of restricting the analysis to studies additionally adjusting for HCV coinfection, HIV coinfection and antiviral treatment in addition to age and sex. All included studies were cohort or nested case-control studies reporting Hazards Ratios.

HR, hazard ratio; CI, confidence interval; DM, diabetes mellitus.

**
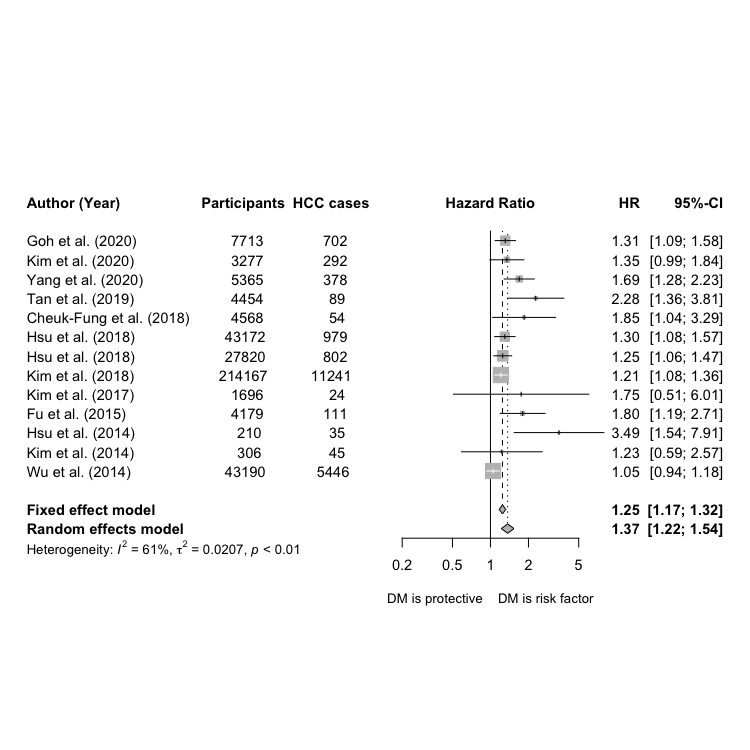
**

**Figure S3.** Sensitivity analysis of primary meta-analysis demonstrating the influence of restricting the analysis to studies additionally adjusting for cirrhosis in addition to age and sex. All included studies were cohort or nested case-control studies reporting Hazards Ratios.

HR, hazard ratio; CI, confidence interval; DM, diabetes mellitus.


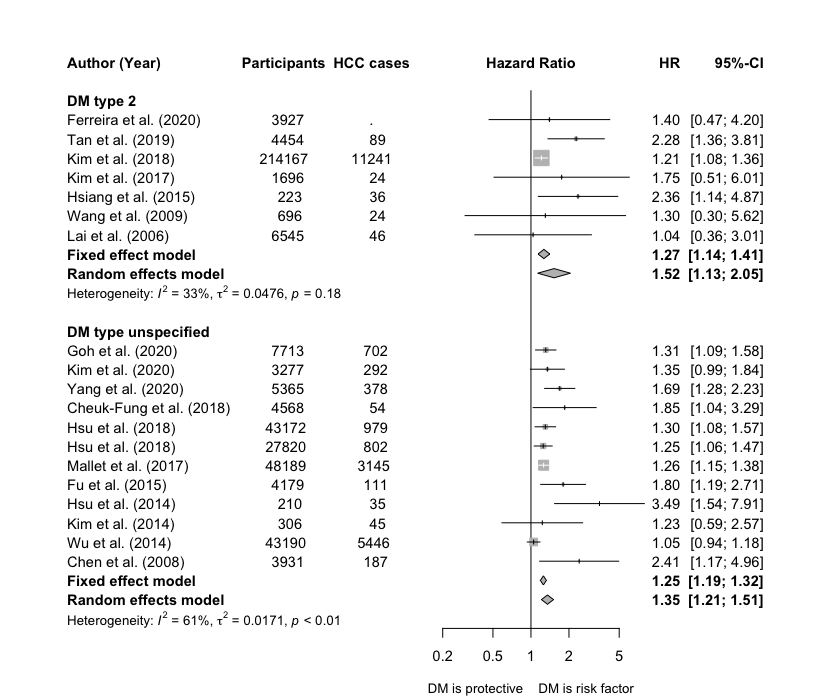


**Figure S4.** Sensitivity analysis of primary meta-analysis stratifying studies by diabetes mellitus disease subtype. All included studies were cohort or nested case-control studies reporting Hazards Ratios.

HR, hazard ratio; CI, confidence interval; DM, diabetes mellitus.


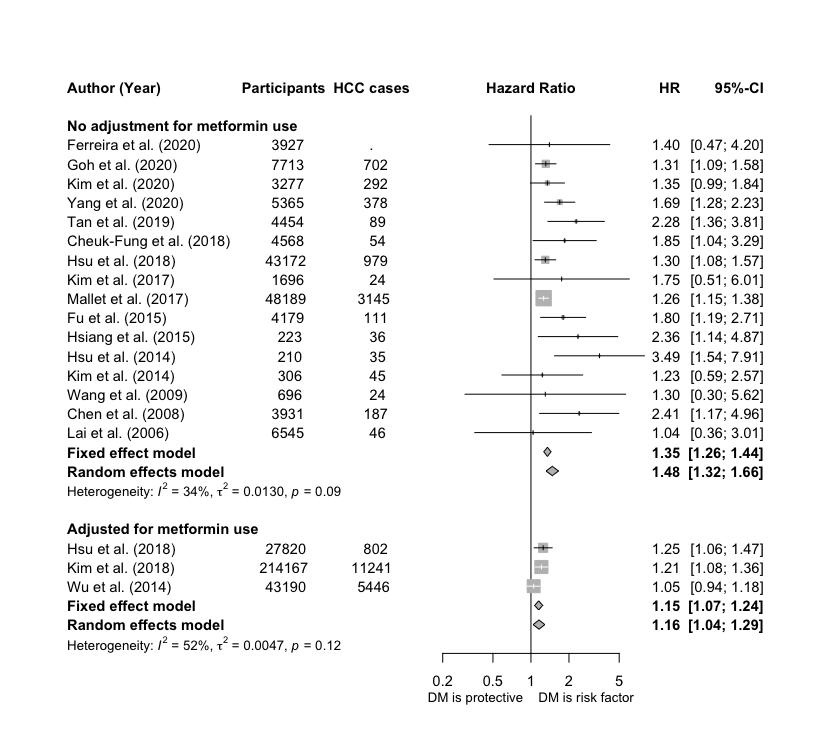
 **Figure S5**. Sensitivity analysis restricting the primary meta-analysis to studies adjusting for use of metformin. All included studies were cohort or nested case-control studies reporting Hazards Ratios.

HR, hazard ratio; CI, confidence interval; DM, diabetes mellitus.

**References for supplement**

1. Ferreira G, Stuurman AL, Horsmans Y, et al. Hepatitis B virus infection and the risk of liver disease progression in type 2 diabetic patients with potential nonalcoholic fatty liver disease. *Eur J Gastroenterol Hepatol*. 2020;32(1):101-109. doi:10.1097/MEG.0000000000001537

2. Goh MJ, Sinn DH, Kim S, et al. Statin Use and the Risk of Hepatocellular Carcinoma in Patients With Chronic Hepatitis B. *Hepatology*. 2020;71(6):2023-2032. doi:10.1002/hep.30973

3. Kim SU, Seo YS, Lee HA, et al. Validation of the CAMD Score in Patients With Chronic Hepatitis B Virus Infection Receiving Antiviral Therapy. *Clin Gastroenterol Hepatol*. 2020;18(3):693-699.e1. doi:10.1016/j.cgh.2019.06.028

4. Lim CT, Goh GBB, Li H, et al. Presence of Hepatic Steatosis Does Not Increase the Risk of Hepatocellular Carcinoma in Patients With Chronic Hepatitis B Over Long Follow-Up. *Microbiol Insights*. 2020;13:117863612091887. doi:10.1177/1178636120918878

5. Yang HI, Yeh ML, Wong GL et al. Real-World Effectiveness From the Asia Pacific Rim Liver Consortium for HBV Risk Score for the Prediction of Hepatocellular Carcinoma in Chronic Hepatitis B Patients Treated With Oral Antiviral Therapy - PubMed. *J Infect Dis*. 2020;221(3):389-399. https://pubmed.ncbi.nlm.nih.gov/31550363/. Accessed July 1, 2020.

6. Yip TC, Wong VW, Chan HL, et al. Thiazolidinediones reduce the risk of hepatocellular carcinoma and hepatic events in diabetic patients with chronic hepatitis B. *J Viral Hepat*. May 2020:jvh.13307. doi:10.1111/jvh.13307

7. Brichler S, Nahon P, Zoulim F, et al. Non-virological factors are drivers of hepatocellular carcinoma in virosuppressed hepatitis B cirrhosis: Results of ANRS CO12 CirVir cohort. *J Viral Hepat*. 2019;26(3):384-396. doi:10.1111/jvh.13029

8. Cho H, Chang Y, Lee JH, et al. Radiologic Nonalcoholic Fatty Liver Disease Increases the Risk of Hepatocellular Carcinoma in Patients with Suppressed Chronic Hepatitis B. *J Clin Gastroenterol*. 2019. doi:10.1097/MCG.0000000000001217

9. Shyu Y, Huang T, Chien C, Yeh C, Lin C, Chien R. Diabetes poses a higher risk of hepatocellular carcinoma and mortality in patients with chronic hepatitis B: A population‐based cohort study. *J Viral Hepat*. 2019;26(6):718-726. doi:10.1111/jvh.13077

10. Deyo RA, Cherkin DC, Ciol MA. Adapting a clinical comorbidity index for use with ICD-9-CM administrative databases. *J Clin Epidemiol*. 1992;45(6):613-619. doi:10.1016/0895-4356(92)90133-8

11. Tan Y, Zhang X, Zhang W, et al. The influence of metabolic syndrome on the risk of hepatocellular carcinoma in patients with chronic hepatitis B infection in mainland China. *Cancer Epidemiol Biomarkers Prev*. 2019;28(12):2038-2046. doi:10.1158/1055-9965.EPI-19-0303

12. Wang H-W, Lai H-C, Hu T-H, et al. Stratification of hepatocellular carcinoma risk through modified FIB-4 index in chronic hepatitis B patients on entecavir therapy. *J Gastroenterol Hepatol*. 2019;34(2):442-449. doi:10.1111/jgh.14372

13. Hsu YC, Yip TCF, Ho HJ, et al. Development of a scoring system to predict hepatocellular carcinoma in Asians on antivirals for chronic hepatitis B. *J Hepatol*. 2018;69(2):278-285. doi:10.1016/j.jhep.2018.02.032

14. Hsu YC, Ho HJ, Lee TY, et al. Temporal trend and risk determinants of hepatocellular carcinoma in chronic hepatitis B patients on entecavir or tenofovir. *J Viral Hepat*. 2018;25(5):543-551. doi:10.1111/jvh.12832

15. Kennedy K, Graham SM, Arora N, Shuhart MC, Kim HN. Hepatocellular carcinoma among US and non- US-born patients with chronic hepatitis B: Risk factors and age at diagnosis. *PLoS One*. 2018;13(9). doi:10.1371/journal.pone.0204031

16. Kim K, Choi S, Park SM. Association of fasting serum glucose level and type 2 diabetes with hepatocellular carcinoma in men with chronic hepatitis B infection: A large cohort study. *Eur J Cancer*. 2018;102:103-113. doi:10.1016/j.ejca.2018.07.008

17. Li X, Xu H, Gao P. Diabetes Mellitus is a Risk Factor for Hepatocellular Carcinoma in Patients with Chronic Hepatitis B Virus Infection in China. *Med Sci Monit*. 2018;24:6729-6734. doi:10.12659/MSM.911702

18. Cheuk-Fung Yip T, Wai-Sun Wong V, Lik-Yuen Chan H, et al. Effects of Diabetes and Glycemic Control on Risk of Hepatocellular Carcinoma After Seroclearance of Hepatitis B Surface Antigen. *Clin Gastroenterol Hepatol*. 2018;16(5):765-773.e2. doi:10.1016/j.cgh.2017.12.009

19. Chan AWH, Wong GLH, Chan H-Y, et al. Concurrent fatty liver increases risk of hepatocellular carcinoma among patients with chronic hepatitis B. *J Gastroenterol Hepatol*. 2017;32(3):667-676. doi:10.1111/jgh.13536

20. Chayanupatkul M, Omino R, Mittal S, et al. Hepatocellular carcinoma in the absence of cirrhosis in patients with chronic hepatitis B virus infection. *J Hepatol*. 2017;66(2):355-362. doi:10.1016/j.jhep.2016.09.013

21. Han H, Deng H, Han T, Zhao H, Hou F, Qi X. Association between hepatocellular carcinoma and type 2 diabetes mellitus in chinese hepatitis b virus cirrhosis patients: A case-control study. *Med Sci Monit*. 2017;23:3324-3334. doi:10.12659/MSM.902440

22. Kim JH, Sinn DH, Gwak G-Y, et al. Insulin resistance and the risk of hepatocellular carcinoma in chronic hepatitis B patients. *J Gastroenterol Hepatol*. 2017;32(5):1100-1106. doi:10.1111/jgh.13647

23. Mallet V, Hamed K, Schwarzinger M. Prognosis of patients with chronic hepatitis B in France (2008–2013): A nationwide, observational and hospital-based study. *J Hepatol*. 2017;66(3):514-520. doi:10.1016/j.jhep.2016.10.031

24. Shim JJ, Oh CH, Kim JW, Lee CK, Kim BH. Liver cirrhosis stages and the incidence of hepatocellular carcinoma in chronic hepatitis B patients receiving antiviral therapy. *Scand J Gastroenterol*. 2017;52(9):1029-1036. doi:10.1080/00365521.2017.1335773

25. Yu MW, Lin CL, Liu CJ, Yang SH, Tseng YL, Wu CF. Influence of Metabolic Risk Factors on Risk of Hepatocellular Carcinoma and Liver-Related Death in Men With Chronic Hepatitis B: A Large Cohort Study. *Gastroenterology*. 2017;153(4):1006-1017.e5. doi:10.1053/j.gastro.2017.07.001

26. Lee J, Yoo SH, Sohn W, et al. Obesity and hepatocellular carcinoma in patients receiving entecavir for chronic Hepatitis B. *Clin Mol Hepatol*. 2016;22(3):339-349. doi:10.3350/cmh.2016.0021

27. Brouwer WP, van der Meer AJ, Boonstra A, et al. The impact of PNPLA3 (rs738409 C>G) polymorphisms on liver histology and long-term clinical outcome in chronic hepatitis B patients. *Liver Int*. 2015;35(2):438-447. doi:10.1111/liv.12695

28. Fu S-C, Huang Y-W, Wang T-C, Hu J-T, Chen D-S, Yang S-S. Increased risk of hepatocellular carcinoma in chronic hepatitis B patients with new onset diabetes: a nationwide cohort study. *Aliment Pharmacol Ther*. 2015;41(11):1200-1209. doi:10.1111/apt.13191

29. Hsiang JC, Gane EJ, Bai WW, Gerred SJ. Type 2 diabetes: A risk factor for liver mortality and complications in hepatitis B cirrhosis patients. *J Gastroenterol Hepatol*. 2015;30(3):591-599. doi:10.1111/jgh.12790

30. Hsu YC, Wu CY, Lane HY, et al. Determinants of hepatocellular carcinoma in cirrhotic patients treated with nucleos(t)ide analogues for chronic hepatitis B. *J Antimicrob Chemother*. 2014;69(7):1920-1927. doi:10.1093/jac/dku041

31. Kim SS, Hwang JC, Lim SG, Ahn SJ, Cheong JY, Cho SW. Effect of Virological Response to Entecavir on the Development of Hepatocellular Carcinoma in Hepatitis B Viral Cirrhotic Patients: Comparison Between Compensated and Decompensated Cirrhosis. *Am J Gastroenterol*. 2014;109(8):1223-1233. doi:10.1038/ajg.2014.145

32. Wu CY, Lin JT, Ho HJ, et al. Association of nucleos(T)ide analogue therapy with reduced risk of hepatocellular carcinoma in patients with chronic hepatitis B - A nationwide cohort study. *Gastroenterology*. 2014;147(1). doi:10.1053/j.gastro.2014.03.048

33. Chen CT, Chen JY, Wang JH, et al. Diabetes mellitus, metabolic syndrome and obesity are not significant risk factors for hepatocellular carcinoma in an HBV- and HCV-endemic area of Southern Taiwan. *Kaohsiung J Med Sci*. 2013;29(8):451-459. doi:10.1016/j.kjms.2012.12.006

34. Gao C, Fang L, Zhao HC, Li JT, Yao SK. Potential role of diabetes mellitus in the progression of cirrhosis to hepatocellular carcinoma: A cross-sectional case-control study from Chinese patients with HBV infection. *Hepatobiliary Pancreat Dis Int*. 2013;12(4):385-393. doi:10.1016/S1499-3872(13)60060-0

35. Li Q, Li W-W, Yang X, et al. Type 2 diabetes and hepatocellular carcinoma: A case-control study in patients with chronic hepatitis B. *Int J Cancer*. 2012;131(5):1197-1202. doi:10.1002/ijc.27337

36. Chao LT, Wu CF, Sung FY, et al. Insulin, glucose and hepatocellular carcinoma risk in male hepatitis B carriers: Results from 17-year follow-up of a population-based cohort. *Carcinogenesis*. 2011;32(6):876-881. doi:10.1093/carcin/bgr058

37. Walter SR, Thein H-H, Gidding HF, et al. Risk factors for hepatocellular carcinoma in a cohort infected with hepatitis B or C. *J Gastroenterol Hepatol*. 2011;26(12):1757-1764. doi:10.1111/j.1440-1746.2011.06785.x

38. Wang CS, Yao WJ, Chang TT, Wang ST, Chou P. The impact of type 2 diabetes on the development of hepatocellular carcinoma in different viral hepatitis statuses. *Cancer Epidemiol Biomarkers Prev*. 2009;18(7):2054-2060. doi:10.1158/1055-9965.EPI-08-1131

39. Chen CL, Yang HI, Yang WS, et al. Metabolic Factors and Risk of Hepatocellular Carcinoma by Chronic Hepatitis B/C Infection: A Follow-up Study in Taiwan. *Gastroenterology*. 2008;135(1):111-121. doi:10.1053/j.gastro.2008.03.073

40. Yu MW, Shih WL, Lin CL, et al. Body-mass index and progression of hepatitis B: A population-based cohort study in men. *J Clin Oncol*. 2008;26(34):5576-5582. doi:10.1200/JCO.2008.16.1075

41. Lai MS, Hsieh MS, Chiu YH, Chen THH. Type 2 diabetes and hepatocellular carcinoma: A cohort study in high prevalence area of hepatitis virus infection. *Hepatology*. 2006;43(6):1295-1302. doi:10.1002/hep.21208
